# 3-{2-[(3-Phenyl­quinoxalin-2-yl)­oxy]ethyl}-1,3-oxazolidin-2-one

**DOI:** 10.1107/S1600536811014632

**Published:** 2011-04-29

**Authors:** Ballo Daouda, Lydia Brelot, Mouhamadou Lamine Doumbia, El Mokhtar Essassi, Seik Weng Ng

**Affiliations:** a Laboratoire de Chimie Organique Hétérocyclique, Pôle de Compétences Pharmacochimie, Université Mohammed V-Agdal, BP 1014 Avenue Ibn Batout, Rabat, Morocco; b Service de Radiocristallographie, Institut de Chimie UMR 7177, CNRS-UdS Tour de Chimie, Rez-de-chaussée 1 rue Blaise Pascal, BP 296/R867008, Strasbourg Cedex, France; c Department of Chemistry, University of Malaya, 50603 Kuala Lumpur, Malaysia

## Abstract

The asymmetric unit of the title compound, C_19_H_17_N_3_O_3_, consists of two independent mol­ecules that are disposed about a pseudo-centre of inversion. The plane of the phenyl substituent is twisted by 38.1 (1)° [43.6 (1)° in the second mol­ecule] out of the plane of the quinoxaline ring system. The five-membered ring of the substituent at the 2-position adopts an envelope conformation; the 5-CH_2_ atom representing the flap lies out of the plane defined by the other four atoms [deviation 0.264 (7) Å in the first mol­ecule and 0.291 (6) Å in the second]. The dihedral angle between the five-membered ring and the 4-phenyl ring is 84.9 (1)° while that between the five-membered ring and the 5-phenyl ring is 65.6 (1)°.

## Related literature

For the crystal structure of 3-[2-(3-methyl­quinoxalin-2-yl­oxy)eth­yl]-1,3-oxazolidin-2-one, see: Ahoya *et al.* (2010 ▶).
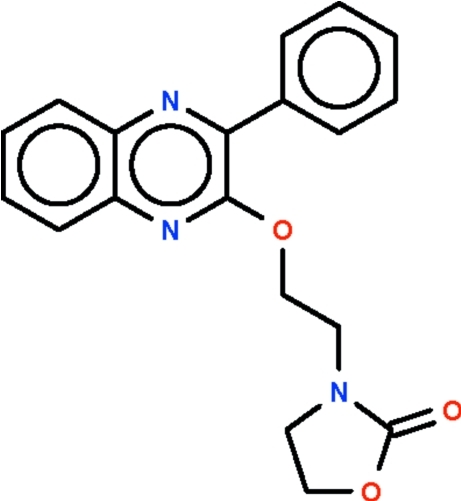


## Experimental

### 

#### Crystal data

C_19_H_17_N_3_O_3_
*M* *_r_* = 335.36
Orthorhombic, 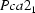

*a* = 10.1392 (4) Å
*b* = 9.1398 (4) Å
*c* = 34.8462 (15) Å
*V* = 3229.2 (2) Å^3^
*Z* = 8
Mo *K*α radiation
μ = 0.10 mm^−1^
*T* = 173 K
0.40 × 0.35 × 0.30 mm

#### Data collection

Bruker X8 APEXII diffractometer
25550 measured reflections
4767 independent reflections
4101 reflections with *I* > 2σ(*I*)
*R*_int_ = 0.029

#### Refinement

*R*[*F* ^2^ > 2σ(*F* ^2^)] = 0.047
*wR*(*F* ^2^) = 0.123
*S* = 1.03
4767 reflections
451 parameters
1 restraint
H-atom parameters constrained
Δρ_max_ = 0.32 e Å^−3^
Δρ_min_ = −0.21 e Å^−3^
Absolute structure: 4149 Friedel pairs merged

### 

Data collection: *APEX2* (Bruker, 2008 ▶); cell refinement: *SAINT* (Bruker, 2008 ▶); data reduction: *SAINT*; program(s) used to solve structure: *SHELXS97* (Sheldrick, 2008 ▶); program(s) used to refine structure: *SHELXL97* (Sheldrick, 2008 ▶); molecular graphics: *X-SEED* (Barbour, 2001 ▶); software used to prepare material for publication: *publCIF* (Westrip, 2010 ▶).

## Supplementary Material

Crystal structure: contains datablocks global, I. DOI: 10.1107/S1600536811014632/bt5521sup1.cif

Structure factors: contains datablocks I. DOI: 10.1107/S1600536811014632/bt5521Isup2.hkl

Additional supplementary materials:  crystallographic information; 3D view; checkCIF report

## Figures and Tables

**Fig. 1.**
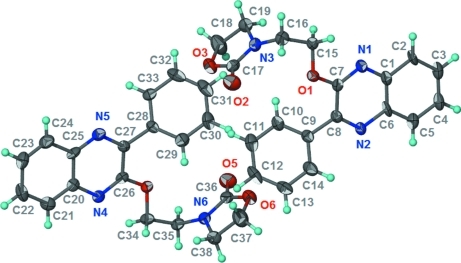
Anisotropic displacement ellipsoid plot (Barbour, 2001) of two independent molecules of C19H17N3O3 disposed about a pseudo center-of-inversion at the 70% probability level; H atoms are drawn as arbitrary radius.

## supplementary crystallographic information

### Comment

A recent study reports the crystal structure of 3-[2-(3-methylquinoxalin-2-yloxy)ethyl]-1,3-oxazolidin-2-one (Ahoya *et al.*, 2010). The present study reports the 3-phenyl analog (Scheme I). The asymmetric unit consists of two independent molecules that are disposed about a pseudo center-of-inversion (Fig. 1). The phenyl substituent of the 3-position of the quinoxaline ring is twisted by 38.1 (1)° [43.6 (1)° in the second molecule]. The five-membering ring of the substituent at the 2-position adopts the shape of an envelope; the 5-CH_2_ atom representing the flap lies out of the plane defined by the other four atoms [deviation 0.264 (7) Å in the first molecule and 0.291 (6) Å in the second molecule].

### Experimental

3-Phenyl-quinoxalin-2-one (1.25 mmol, 0.28 g), di(chloroethyl)amine hydrochloride (2.66 mmol, 0.50 g), potassium carbonate (4.00 mmol, 0.52 g) and tetra-*n*-butylammonium bromide (0.01 mmol, 0.003 g) were heated in DMF (40 ml); the progress of the reaction was monitored by thin layer chromatography. On completion, the solvent was removed under reduced pressure and the residue was chromatographed on silica column (*n*-hexane/ethyl acetate:4/6). Recrystallization was effected by using the sample solvent system.

### Refinement

H atoms were placed in calculated positions (C—H 0.95–0.99 Å) and were included in the refinement in the riding model approximation, with *U*(H) set to 1.2*U*_eq_(C); 4149 Friedel pairs were merged.

### Crystal data

**Table:**

C_19_H_17_N_3_O_3_	*F*(000) = 1408
*M**_r_* = 335.36	*D*_x_ = 1.380 Mg m^−^^3^
Orthorhombic, *P**c**a*2_1_	Mo *K*α radiation, λ = 0.71073 Å
Hall symbol: P 2c -2ac	Cell parameters from 9872 reflections
*a* = 10.1392 (4) Å	θ = 2.3–30.0°
*b* = 9.1398 (4) Å	µ = 0.10 mm^−^^1^
*c* = 34.8462 (15) Å	*T* = 173 K
*V* = 3229.2 (2) Å^3^	Prism, colourless
*Z* = 8	0.40 × 0.35 × 0.30 mm

### Data collection

**Table:**

Bruker X8 APEXII diffractometer	4101 reflections with *I* > 2σ(*I*)
Radiation source: fine-focus sealed tube	*R*_int_ = 0.029
graphite	θ_max_ = 30.0°, θ_min_ = 2.5°
φ and ω scans	*h* = −14→14
25550 measured reflections	*k* = −12→12
4767 independent reflections	*l* = −49→47

### Refinement

**Table:**

Refinement on *F*^2^	Secondary atom site location: difference Fourier map
Least-squares matrix: full	Hydrogen site location: inferred from neighbouring sites
*R*[*F*^2^ > 2σ(*F*^2^)] = 0.047	H-atom parameters constrained
*wR*(*F*^2^) = 0.123	*w* = 1/[σ^2^(*F*_o_^2^) + (0.0525*P*)^2^ + 1.9526*P*] where *P* = (*F*_o_^2^ + 2*F*_c_^2^)/3
*S* = 1.03	(Δ/σ)_max_ = 0.001
4767 reflections	Δρ_max_ = 0.32 e Å^−^^3^
451 parameters	Δρ_min_ = −0.21 e Å^−^^3^
1 restraint	Absolute structure: 4149 Friedel pairs merged
Primary atom site location: structure-invariant direct methods	

### Fractional atomic coordinates and isotropic or equivalent isotropic displacement parameters (Å^2^)

**Table:**

	*x*	*y*	*z*	*U*_iso_*/*U*_eq_	
O1	0.3755 (2)	0.6241 (2)	0.49991 (7)	0.0238 (4)	
O2	0.1310 (2)	0.3976 (3)	0.55878 (9)	0.0433 (6)	
O3	0.2900 (3)	0.3586 (3)	0.60256 (7)	0.0380 (6)	
O4	0.3780 (2)	0.8711 (2)	0.79097 (7)	0.0236 (5)	
O5	0.6231 (2)	1.1037 (3)	0.73247 (9)	0.0426 (6)	
O6	0.4633 (2)	1.1405 (3)	0.68853 (7)	0.0378 (6)	
N1	0.5295 (2)	0.7359 (3)	0.46162 (7)	0.0231 (5)	
N2	0.4634 (3)	1.0015 (3)	0.49854 (11)	0.0229 (7)	
N3	0.3471 (2)	0.3614 (3)	0.54162 (8)	0.0251 (5)	
N4	0.2237 (2)	0.7592 (3)	0.82911 (7)	0.0218 (5)	
N5	0.2891 (3)	0.4944 (3)	0.79247 (12)	0.0257 (7)	
N6	0.4059 (2)	1.1361 (3)	0.74966 (8)	0.0233 (5)	
C1	0.5889 (3)	0.8656 (4)	0.45148 (9)	0.0238 (7)	
C2	0.6894 (3)	0.8632 (4)	0.42211 (8)	0.0264 (7)	
H2	0.7162	0.7739	0.4105	0.032*	
C3	0.7450 (5)	0.9938 (4)	0.41148 (15)	0.0316 (10)	
H3	0.8122	0.9931	0.3925	0.038*	
C4	0.7080 (3)	1.1262 (5)	0.42711 (10)	0.0307 (8)	
H4	0.7466	1.2147	0.4183	0.037*	
C5	0.6123 (4)	1.1283 (4)	0.45641 (10)	0.0293 (7)	
H5	0.5876	1.2180	0.4681	0.035*	
C6	0.5546 (3)	0.9977 (3)	0.46791 (10)	0.0232 (8)	
C7	0.4435 (3)	0.7436 (3)	0.48952 (8)	0.0208 (5)	
C8	0.4129 (2)	0.8775 (3)	0.50968 (8)	0.0205 (5)	
C9	0.3280 (3)	0.8803 (3)	0.54447 (8)	0.0221 (5)	
C10	0.3353 (3)	0.7709 (4)	0.57228 (8)	0.0268 (6)	
H10	0.3893	0.6875	0.5681	0.032*	
C11	0.2637 (3)	0.7835 (4)	0.60623 (9)	0.0333 (7)	
H11	0.2688	0.7088	0.6251	0.040*	
C12	0.1847 (3)	0.9053 (4)	0.61239 (9)	0.0354 (8)	
H12	0.1376	0.9154	0.6358	0.042*	
C13	0.1748 (4)	1.0128 (4)	0.58411 (14)	0.0321 (9)	
H13	0.1169	1.0935	0.5875	0.038*	
C14	0.2494 (5)	1.0014 (3)	0.55127 (15)	0.0298 (10)	
H14	0.2469	1.0781	0.5329	0.036*	
C15	0.4161 (4)	0.4879 (3)	0.48313 (12)	0.0251 (8)	
H15A	0.5103	0.4689	0.4888	0.030*	
H15B	0.4044	0.4907	0.4549	0.030*	
C16	0.3308 (3)	0.3702 (4)	0.50051 (10)	0.0271 (7)	
H16A	0.2372	0.3907	0.4945	0.033*	
H16B	0.3539	0.2747	0.4889	0.033*	
C17	0.2459 (3)	0.3749 (3)	0.56612 (10)	0.0295 (6)	
C18	0.4324 (4)	0.3412 (6)	0.60200 (11)	0.0493 (10)	
H18A	0.4597	0.2588	0.6186	0.059*	
H18B	0.4766	0.4315	0.6109	0.059*	
C19	0.4661 (3)	0.3101 (4)	0.56046 (9)	0.0269 (6)	
H19A	0.5447	0.3657	0.5520	0.032*	
H19B	0.4808	0.2044	0.5559	0.032*	
C20	0.1637 (3)	0.6307 (4)	0.84002 (9)	0.0209 (6)	
C21	0.0673 (3)	0.6295 (4)	0.86756 (11)	0.0289 (7)	
H21	0.0413	0.7194	0.8790	0.035*	
C22	0.0066 (5)	0.5014 (4)	0.87928 (13)	0.0317 (11)	
H22	−0.0590	0.5017	0.8988	0.038*	
C23	0.0464 (4)	0.3684 (4)	0.86090 (11)	0.0327 (8)	
H23	0.0046	0.2795	0.8681	0.039*	
C24	0.1396 (4)	0.3652 (4)	0.83418 (13)	0.0319 (8)	
H24	0.1646	0.2742	0.8232	0.038*	
C25	0.2034 (4)	0.4973 (3)	0.82158 (12)	0.0237 (8)	
C26	0.3085 (3)	0.7517 (3)	0.80137 (8)	0.0205 (5)	
C27	0.3400 (3)	0.6188 (3)	0.78065 (8)	0.0215 (5)	
C28	0.4249 (3)	0.6163 (3)	0.74596 (8)	0.0216 (5)	
C29	0.4178 (3)	0.7269 (4)	0.71840 (9)	0.0277 (6)	
H29	0.3636	0.8102	0.7227	0.033*	
C30	0.4903 (3)	0.7147 (4)	0.68470 (9)	0.0333 (7)	
H30	0.4852	0.7897	0.6659	0.040*	
C31	0.5706 (3)	0.5932 (4)	0.67837 (10)	0.0352 (7)	
H31	0.6195	0.5855	0.6552	0.042*	
C32	0.5793 (5)	0.4847 (4)	0.70545 (15)	0.0358 (10)	
H32	0.6336	0.4017	0.7009	0.043*	
C33	0.5080 (4)	0.4961 (3)	0.73990 (14)	0.0237 (9)	
H33	0.5162	0.4225	0.7590	0.028*	
C34	0.3381 (4)	1.0076 (3)	0.80828 (14)	0.0258 (8)	
H34A	0.3516	1.0042	0.8364	0.031*	
H34B	0.2435	1.0265	0.8032	0.031*	
C35	0.4223 (3)	1.1268 (3)	0.79058 (9)	0.0225 (6)	
H35A	0.3987	1.2220	0.8023	0.027*	
H35B	0.5162	1.1072	0.7965	0.027*	
C36	0.5075 (3)	1.1237 (3)	0.72500 (10)	0.0283 (6)	
C37	0.3211 (4)	1.1544 (5)	0.68928 (11)	0.0441 (9)	
H37A	0.2787	1.0623	0.6808	0.053*	
H37B	0.2918	1.2347	0.6722	0.053*	
C38	0.2867 (3)	1.1876 (3)	0.73069 (9)	0.0264 (6)	
H38A	0.2724	1.2936	0.7349	0.032*	
H38B	0.2078	1.1327	0.7392	0.032*	

### Atomic displacement parameters (Å^2^)

**Table:**

	*U*^11^	*U*^22^	*U*^33^	*U*^12^	*U*^13^	*U*^23^
O1	0.0227 (10)	0.0214 (10)	0.0272 (10)	0.0002 (9)	0.0067 (8)	−0.0008 (8)
O2	0.0246 (10)	0.0476 (15)	0.0577 (16)	0.0079 (10)	0.0092 (11)	0.0058 (13)
O3	0.0388 (13)	0.0437 (14)	0.0314 (12)	0.0074 (11)	0.0109 (10)	0.0041 (11)
O4	0.0293 (11)	0.0163 (9)	0.0252 (10)	−0.0016 (8)	0.0032 (9)	−0.0011 (8)
O5	0.0239 (11)	0.0443 (14)	0.0596 (17)	0.0070 (10)	0.0094 (11)	0.0058 (13)
O6	0.0382 (13)	0.0409 (14)	0.0342 (12)	0.0083 (11)	0.0118 (10)	0.0039 (11)
N1	0.0240 (10)	0.0263 (12)	0.0190 (10)	0.0006 (9)	0.0022 (8)	0.0018 (9)
N2	0.0232 (16)	0.0223 (15)	0.0231 (15)	−0.0025 (8)	0.0064 (13)	0.0012 (9)
N3	0.0206 (10)	0.0283 (13)	0.0263 (12)	0.0009 (9)	0.0006 (9)	0.0047 (10)
N4	0.0238 (11)	0.0215 (11)	0.0201 (10)	−0.0001 (9)	−0.0031 (8)	−0.0013 (9)
N5	0.0267 (17)	0.0215 (16)	0.0289 (17)	0.0000 (9)	−0.0015 (14)	−0.0010 (9)
N6	0.0191 (10)	0.0226 (11)	0.0281 (12)	0.0002 (9)	0.0021 (9)	0.0030 (10)
C1	0.0219 (13)	0.0323 (17)	0.0173 (12)	−0.0003 (12)	0.0013 (10)	0.0039 (12)
C2	0.0294 (14)	0.0388 (18)	0.0109 (11)	−0.0004 (13)	0.0079 (11)	−0.0013 (12)
C3	0.029 (2)	0.041 (3)	0.026 (2)	−0.0077 (12)	0.0040 (16)	0.0064 (12)
C4	0.0256 (15)	0.0408 (19)	0.0256 (15)	−0.0087 (14)	0.0061 (12)	0.0095 (14)
C5	0.0314 (16)	0.0314 (17)	0.0251 (15)	−0.0041 (14)	0.0011 (12)	0.0051 (14)
C6	0.0150 (13)	0.041 (2)	0.0132 (14)	−0.0022 (10)	0.0051 (12)	0.0045 (10)
C7	0.0210 (11)	0.0216 (13)	0.0199 (12)	−0.0006 (10)	−0.0003 (9)	0.0022 (10)
C8	0.0177 (11)	0.0246 (13)	0.0191 (11)	0.0001 (10)	0.0002 (9)	0.0003 (10)
C9	0.0193 (11)	0.0236 (13)	0.0234 (12)	−0.0032 (10)	0.0016 (9)	−0.0031 (11)
C10	0.0263 (13)	0.0334 (15)	0.0207 (12)	−0.0014 (12)	0.0007 (10)	0.0001 (12)
C11	0.0333 (15)	0.0439 (18)	0.0227 (13)	−0.0083 (14)	−0.0002 (11)	0.0020 (14)
C12	0.0290 (15)	0.052 (2)	0.0250 (14)	−0.0129 (14)	0.0094 (12)	−0.0111 (14)
C13	0.0248 (17)	0.0318 (18)	0.040 (2)	−0.0047 (12)	0.0084 (16)	−0.0117 (14)
C14	0.0236 (17)	0.032 (2)	0.034 (3)	−0.0045 (11)	0.0093 (15)	−0.0036 (12)
C15	0.035 (2)	0.0204 (16)	0.0197 (17)	0.0014 (11)	0.0060 (14)	−0.0005 (10)
C16	0.0282 (15)	0.0248 (15)	0.0283 (14)	−0.0025 (12)	−0.0027 (12)	0.0000 (13)
C17	0.0297 (14)	0.0204 (13)	0.0384 (16)	0.0025 (12)	0.0078 (12)	0.0047 (13)
C18	0.0375 (18)	0.082 (3)	0.0287 (17)	0.0145 (19)	0.0006 (14)	0.0080 (19)
C19	0.0222 (12)	0.0308 (15)	0.0278 (13)	0.0016 (11)	−0.0015 (11)	0.0009 (12)
C20	0.0219 (13)	0.0229 (14)	0.0180 (12)	−0.0011 (11)	−0.0073 (10)	−0.0006 (11)
C21	0.0255 (14)	0.0301 (16)	0.0311 (16)	0.0009 (13)	−0.0028 (12)	−0.0037 (14)
C22	0.027 (2)	0.051 (3)	0.0172 (18)	−0.0041 (13)	−0.0003 (14)	0.0041 (12)
C23	0.0376 (17)	0.0300 (17)	0.0305 (17)	−0.0089 (14)	−0.0037 (14)	0.0071 (15)
C24	0.0309 (16)	0.0213 (16)	0.043 (2)	−0.0030 (13)	0.0021 (14)	0.0012 (15)
C25	0.0278 (17)	0.0116 (14)	0.032 (2)	−0.0009 (9)	−0.0067 (15)	0.0007 (9)
C26	0.0214 (11)	0.0179 (12)	0.0223 (12)	0.0000 (10)	−0.0047 (9)	−0.0006 (10)
C27	0.0219 (12)	0.0196 (12)	0.0229 (12)	−0.0001 (10)	0.0015 (9)	−0.0013 (10)
C28	0.0194 (11)	0.0249 (13)	0.0205 (12)	−0.0026 (10)	0.0016 (9)	−0.0041 (11)
C29	0.0263 (13)	0.0281 (14)	0.0287 (14)	0.0001 (12)	−0.0017 (11)	0.0003 (12)
C30	0.0342 (15)	0.0446 (19)	0.0210 (13)	−0.0078 (14)	0.0007 (11)	0.0033 (14)
C31	0.0323 (16)	0.046 (2)	0.0279 (15)	−0.0129 (14)	0.0085 (12)	−0.0145 (14)
C32	0.0286 (19)	0.0362 (19)	0.043 (2)	−0.0036 (14)	0.0104 (18)	−0.0176 (16)
C33	0.0234 (17)	0.0167 (16)	0.031 (2)	−0.0016 (9)	−0.0030 (14)	−0.0027 (10)
C34	0.0269 (18)	0.0196 (16)	0.0310 (19)	0.0011 (10)	−0.0013 (15)	−0.0046 (11)
C35	0.0219 (13)	0.0165 (12)	0.0290 (14)	−0.0025 (11)	−0.0013 (11)	−0.0021 (11)
C36	0.0278 (14)	0.0212 (13)	0.0358 (16)	0.0032 (11)	0.0082 (12)	0.0026 (12)
C37	0.0394 (18)	0.064 (3)	0.0284 (16)	0.0063 (17)	−0.0006 (14)	0.0040 (17)
C38	0.0216 (12)	0.0284 (14)	0.0293 (14)	0.0007 (11)	−0.0016 (11)	−0.0013 (12)

### Geometric parameters (Å, °)

**Table:**

O1—C7	1.342 (3)	C13—H13	0.9500
O1—C15	1.436 (4)	C14—H14	0.9500
O2—C17	1.210 (4)	C15—C16	1.508 (5)
O3—C17	1.355 (4)	C15—H15A	0.9900
O3—C18	1.452 (5)	C15—H15B	0.9900
O4—C26	1.349 (4)	C16—H16A	0.9900
O4—C34	1.444 (4)	C16—H16B	0.9900
O5—C36	1.214 (4)	C18—C19	1.514 (5)
O6—C36	1.356 (4)	C18—H18A	0.9900
O6—C37	1.448 (4)	C18—H18B	0.9900
N1—C7	1.308 (4)	C19—H19A	0.9900
N1—C1	1.376 (4)	C19—H19B	0.9900
N2—C8	1.303 (4)	C20—C21	1.370 (5)
N2—C6	1.413 (5)	C20—C25	1.436 (5)
N3—C17	1.341 (4)	C21—C22	1.385 (5)
N3—C16	1.444 (4)	C21—H21	0.9500
N3—C19	1.451 (4)	C22—C23	1.432 (6)
N4—C26	1.295 (4)	C22—H22	0.9500
N4—C20	1.376 (4)	C23—C24	1.327 (6)
N5—C27	1.316 (4)	C23—H23	0.9500
N5—C25	1.336 (5)	C24—C25	1.438 (5)
N6—C36	1.347 (4)	C24—H24	0.9500
N6—C35	1.438 (4)	C26—C27	1.448 (4)
N6—C38	1.456 (4)	C27—C28	1.484 (4)
C1—C6	1.380 (5)	C28—C29	1.397 (4)
C1—C2	1.445 (4)	C28—C33	1.401 (4)
C2—C3	1.372 (5)	C29—C30	1.390 (4)
C2—H2	0.9500	C29—H29	0.9500
C3—C4	1.379 (6)	C30—C31	1.395 (5)
C3—H3	0.9500	C30—H30	0.9500
C4—C5	1.409 (5)	C31—C32	1.372 (6)
C4—H4	0.9500	C31—H31	0.9500
C5—C6	1.389 (5)	C32—C33	1.405 (7)
C5—H5	0.9500	C32—H32	0.9500
C7—C8	1.445 (4)	C33—H33	0.9500
C8—C9	1.487 (4)	C34—C35	1.515 (5)
C9—C14	1.385 (5)	C34—H34A	0.9900
C9—C10	1.394 (4)	C34—H34B	0.9900
C10—C11	1.393 (4)	C35—H35A	0.9900
C10—H10	0.9500	C35—H35B	0.9900
C11—C12	1.388 (5)	C37—C38	1.515 (5)
C11—H11	0.9500	C37—H37A	0.9900
C12—C13	1.396 (6)	C37—H37B	0.9900
C12—H12	0.9500	C38—H38A	0.9900
C13—C14	1.375 (7)	C38—H38B	0.9900
			
C7—O1—C15	116.6 (2)	H18A—C18—H18B	108.8
C17—O3—C18	109.1 (3)	N3—C19—C18	100.6 (3)
C26—O4—C34	116.1 (3)	N3—C19—H19A	111.7
C36—O6—C37	108.8 (2)	C18—C19—H19A	111.7
C7—N1—C1	115.8 (3)	N3—C19—H19B	111.7
C8—N2—C6	117.4 (3)	C18—C19—H19B	111.7
C17—N3—C16	122.6 (3)	H19A—C19—H19B	109.4
C17—N3—C19	112.2 (3)	C21—C20—N4	121.0 (3)
C16—N3—C19	124.2 (3)	C21—C20—C25	120.4 (3)
C26—N4—C20	117.0 (3)	N4—C20—C25	118.5 (3)
C27—N5—C25	118.4 (3)	C20—C21—C22	122.1 (4)
C36—N6—C35	122.6 (3)	C20—C21—H21	119.0
C36—N6—C38	111.9 (3)	C22—C21—H21	119.0
C35—N6—C38	124.4 (2)	C21—C22—C23	117.4 (4)
N1—C1—C6	122.5 (3)	C21—C22—H22	121.3
N1—C1—C2	118.5 (3)	C23—C22—H22	121.3
C6—C1—C2	119.0 (3)	C24—C23—C22	122.2 (4)
C3—C2—C1	117.9 (4)	C24—C23—H23	118.9
C3—C2—H2	121.0	C22—C23—H23	118.9
C1—C2—H2	121.0	C23—C24—C25	121.0 (4)
C2—C3—C4	123.0 (4)	C23—C24—H24	119.5
C2—C3—H3	118.5	C25—C24—H24	119.5
C4—C3—H3	118.5	N5—C25—C20	122.6 (3)
C3—C4—C5	119.1 (4)	N5—C25—C24	120.5 (3)
C3—C4—H4	120.5	C20—C25—C24	116.8 (4)
C5—C4—H4	120.5	N4—C26—O4	120.3 (3)
C6—C5—C4	119.2 (4)	N4—C26—C27	124.3 (3)
C6—C5—H5	120.4	O4—C26—C27	115.4 (2)
C4—C5—H5	120.4	N5—C27—C26	118.8 (3)
C1—C6—C5	121.7 (3)	N5—C27—C28	118.0 (3)
C1—C6—N2	120.0 (3)	C26—C27—C28	123.2 (3)
C5—C6—N2	118.2 (3)	C29—C28—C33	119.7 (3)
N1—C7—O1	120.0 (3)	C29—C28—C27	121.2 (3)
N1—C7—C8	123.4 (3)	C33—C28—C27	119.0 (3)
O1—C7—C8	116.6 (2)	C30—C29—C28	119.7 (3)
N2—C8—C7	120.5 (3)	C30—C29—H29	120.1
N2—C8—C9	117.1 (3)	C28—C29—H29	120.1
C7—C8—C9	122.4 (2)	C29—C30—C31	120.4 (3)
C14—C9—C10	119.0 (3)	C29—C30—H30	119.8
C14—C9—C8	119.1 (3)	C31—C30—H30	119.8
C10—C9—C8	121.6 (3)	C32—C31—C30	120.3 (3)
C9—C10—C11	120.2 (3)	C32—C31—H31	119.9
C9—C10—H10	119.9	C30—C31—H31	119.9
C11—C10—H10	119.9	C31—C32—C33	120.1 (4)
C12—C11—C10	119.9 (3)	C31—C32—H32	120.0
C12—C11—H11	120.1	C33—C32—H32	120.0
C10—C11—H11	120.1	C28—C33—C32	119.8 (4)
C11—C12—C13	119.8 (3)	C28—C33—H33	120.1
C11—C12—H12	120.1	C32—C33—H33	120.1
C13—C12—H12	120.1	O4—C34—C35	107.0 (3)
C14—C13—C12	119.6 (4)	O4—C34—H34A	110.3
C14—C13—H13	120.2	C35—C34—H34A	110.3
C12—C13—H13	120.2	O4—C34—H34B	110.3
C13—C14—C9	121.3 (4)	C35—C34—H34B	110.3
C13—C14—H14	119.3	H34A—C34—H34B	108.6
C9—C14—H14	119.3	N6—C35—C34	112.4 (3)
O1—C15—C16	106.9 (3)	N6—C35—H35A	109.1
O1—C15—H15A	110.3	C34—C35—H35A	109.1
C16—C15—H15A	110.3	N6—C35—H35B	109.1
O1—C15—H15B	110.3	C34—C35—H35B	109.1
C16—C15—H15B	110.3	H35A—C35—H35B	107.9
H15A—C15—H15B	108.6	O5—C36—N6	127.9 (3)
N3—C16—C15	111.9 (3)	O5—C36—O6	122.5 (3)
N3—C16—H16A	109.2	N6—C36—O6	109.6 (3)
C15—C16—H16A	109.2	O6—C37—C38	105.3 (3)
N3—C16—H16B	109.2	O6—C37—H37A	110.7
C15—C16—H16B	109.2	C38—C37—H37A	110.7
H16A—C16—H16B	107.9	O6—C37—H37B	110.7
O2—C17—N3	128.2 (3)	C38—C37—H37B	110.7
O2—C17—O3	122.3 (3)	H37A—C37—H37B	108.8
N3—C17—O3	109.5 (3)	N6—C38—C37	100.1 (3)
O3—C18—C19	105.0 (3)	N6—C38—H38A	111.7
O3—C18—H18A	110.8	C37—C38—H38A	111.7
C19—C18—H18A	110.8	N6—C38—H38B	111.7
O3—C18—H18B	110.8	C37—C38—H38B	111.7
C19—C18—H18B	110.8	H38A—C38—H38B	109.5
			
C7—N1—C1—C6	−3.9 (5)	C26—N4—C20—C21	−176.2 (3)
C7—N1—C1—C2	177.0 (3)	C26—N4—C20—C25	3.1 (4)
N1—C1—C2—C3	178.1 (4)	N4—C20—C21—C22	−179.3 (3)
C6—C1—C2—C3	−1.0 (5)	C25—C20—C21—C22	1.4 (5)
C1—C2—C3—C4	−0.9 (7)	C20—C21—C22—C23	−1.2 (6)
C2—C3—C4—C5	2.5 (7)	C21—C22—C23—C24	1.4 (6)
C3—C4—C5—C6	−2.1 (5)	C22—C23—C24—C25	−1.6 (6)
N1—C1—C6—C5	−177.8 (3)	C27—N5—C25—C20	0.8 (5)
C2—C1—C6—C5	1.3 (5)	C27—N5—C25—C24	176.6 (4)
N1—C1—C6—N2	5.4 (5)	C21—C20—C25—N5	174.5 (3)
C2—C1—C6—N2	−175.5 (3)	N4—C20—C25—N5	−4.9 (5)
C4—C5—C6—C1	0.2 (5)	C21—C20—C25—C24	−1.5 (5)
C4—C5—C6—N2	177.1 (3)	N4—C20—C25—C24	179.2 (3)
C8—N2—C6—C1	−1.2 (5)	C23—C24—C25—N5	−174.4 (4)
C8—N2—C6—C5	−178.1 (4)	C23—C24—C25—C20	1.7 (6)
C1—N1—C7—O1	176.3 (3)	C20—N4—C26—O4	−175.3 (3)
C1—N1—C7—C8	−1.5 (4)	C20—N4—C26—C27	2.2 (4)
C15—O1—C7—N1	9.0 (4)	C34—O4—C26—N4	−8.9 (4)
C15—O1—C7—C8	−173.0 (3)	C34—O4—C26—C27	173.4 (3)
C6—N2—C8—C7	−4.0 (5)	C25—N5—C27—C26	4.4 (5)
C6—N2—C8—C9	173.8 (3)	C25—N5—C27—C28	−174.2 (3)
N1—C7—C8—N2	5.7 (4)	N4—C26—C27—N5	−6.3 (5)
O1—C7—C8—N2	−172.3 (3)	O4—C26—C27—N5	171.2 (3)
N1—C7—C8—C9	−172.0 (3)	N4—C26—C27—C28	172.2 (3)
O1—C7—C8—C9	10.1 (4)	O4—C26—C27—C28	−10.2 (4)
N2—C8—C9—C14	34.6 (4)	N5—C27—C28—C29	140.0 (3)
C7—C8—C9—C14	−147.7 (3)	C26—C27—C28—C29	−38.6 (4)
N2—C8—C9—C10	−138.9 (3)	N5—C27—C28—C33	−36.4 (4)
C7—C8—C9—C10	38.8 (4)	C26—C27—C28—C33	145.1 (3)
C14—C9—C10—C11	0.4 (5)	C33—C28—C29—C30	1.8 (5)
C8—C9—C10—C11	173.9 (3)	C27—C28—C29—C30	−174.4 (3)
C9—C10—C11—C12	0.0 (5)	C28—C29—C30—C31	−0.4 (5)
C10—C11—C12—C13	1.7 (5)	C29—C30—C31—C32	−0.3 (5)
C11—C12—C13—C14	−3.8 (6)	C30—C31—C32—C33	−0.5 (6)
C12—C13—C14—C9	4.3 (7)	C29—C28—C33—C32	−2.7 (5)
C10—C9—C14—C13	−2.6 (6)	C27—C28—C33—C32	173.7 (3)
C8—C9—C14—C13	−176.3 (4)	C31—C32—C33—C28	2.0 (6)
C7—O1—C15—C16	177.1 (3)	C26—O4—C34—C35	−176.2 (3)
C17—N3—C16—C15	123.2 (3)	C36—N6—C35—C34	−123.1 (3)
C19—N3—C16—C15	−69.1 (4)	C38—N6—C35—C34	69.5 (4)
O1—C15—C16—N3	−60.9 (4)	O4—C34—C35—N6	60.0 (4)
C16—N3—C17—O2	−1.2 (5)	C35—N6—C36—O5	0.0 (5)
C19—N3—C17—O2	−170.2 (3)	C38—N6—C36—O5	168.8 (3)
C16—N3—C17—O3	178.6 (3)	C35—N6—C36—O6	−178.5 (3)
C19—N3—C17—O3	9.6 (4)	C38—N6—C36—O6	−9.7 (4)
C18—O3—C17—O2	−176.8 (4)	C37—O6—C36—O5	177.0 (3)
C18—O3—C17—N3	3.4 (4)	C37—O6—C36—N6	−4.4 (4)
C17—O3—C18—C19	−14.1 (4)	C36—O6—C37—C38	15.8 (4)
C17—N3—C19—C18	−17.4 (4)	C36—N6—C38—C37	18.4 (4)
C16—N3—C19—C18	173.9 (3)	C35—N6—C38—C37	−173.1 (3)
O3—C18—C19—N3	18.0 (4)	O6—C37—C38—N6	−19.7 (4)
